# The Impact of COVID-19 Infection in Cancer 2020–2021

**DOI:** 10.3390/cancers14235895

**Published:** 2022-11-29

**Authors:** Gabriella D’Orazi, Mara Cirone

**Affiliations:** 1 Unit of Cellular Networks, Department of Research, IRCCS Regina Elena Cancer National Institute, 00144 Rome, Italy; 2 Department of Neurosciences, Imaging and Clinical Sciences, University G. D’Annunzio, 00131 Chieti, Italy; 3 Department of Experimental Medicine, University of Rome La Sapienza, 00100 Rome, Italy

## Abstract

This Editorial summarizes the findings of the articles submitted in 2020 and 2021 to the Special Issue “The Impact of COVID-19 in Cancer”.

## Introduction

Coronavirus Disease 2019 (COVID-19) is a pandemic of unprecedented, epic proportions caused by highly pathogenic coronavirus SARS-CoV-2 infection and declared as a pandemic by the World Health Organization (WHO) in March 2020. SARS-CoV-2 was reported as a cytopathic virus that induces host cell lysis following infection. SARS-CoV-2 is potentially able to infect angiotensin-converting enzyme 2 (ACE2)-receptor-expressing cells wherever it is [1]. Such infection predominantly affects the lungs, where a massive release of inflammatory cytokines, i.e., the cytokine storm, may contribute to the destruction of infected alveolar cells, fibrosis induction, and endothelial cell damage. The latter promotes the activation of the coagulation pathway with edema, disseminated intravascular coagulation, and coagulopathies, and in some cases leads to acute organ failures [2], which worsen the local clinical picture of the disease with likely systemic impact [3]. Besides producing intense inflammation, these cytokines impair the function of the immune system. Both effects are predisposing factors for cancer onset and may worsen the course of established cancers as well. Cancer patients present a distinct vulnerability to COVID-19 because of their poor health status, concomitant chronic diseases, and immunosuppressive conditions due to cancer itself and antitumor therapies. Since the first case was officially reported in Wuhan (China) in December 2019, COVID-19 has spread rapidly to all countries in the world despite drastic government efforts to contain it. For that reason, it has represented, and still represents, a huge challenge for the health system and the research field looking for molecular mechanisms, drug development, and vaccines. Therefore, understanding the impact of COVID-19 on cancer patients is of pivotal significance for prognostic and therapeutic purpose.

This Special Issue includes 25 original research papers and 10 reviews, commentaries, and perspectives articles collected between 2020 and 2021 from experts in the oncology field, providing the reader with advances in understanding the effects of COVID-19 infection on cancer patients’ survival and treatment.

This issue begins with the concerns about the change in daily practice for oncologic patients due to the pandemic explosion of SARS-CoV-2 infection and the need to convey treatment of all non-deferrable cancer patients to specialized centers (hubs) in order to minimize the risk of infection while maintaining cancer treatment as a priority [4]. This led to the adoption, in the Lombardia region in Italy (where COVID-19 was heavily present since the very beginning), of specific extraordinary measures to decrease the risk of contagion and therefore of cancer progression in such fragile patients, providing a simplified, but complete and easily applicable guide [5]. The authors highlight how the oncologic hub must fulfil some specific requirements such as a high experience in oncologic patient treatment, strict strategies to remain a “COVID-19 free” center, the creation of a dedicated multidisciplinary “hub team”, and dedicated areas for management and treatment of patients who develop COVID-19 symptoms after hospitalization. In the same Lombardia region in Italy, the containment measures performed in ICS Maugeri outpatient clinic (Pavia, Italy) to control infection enabled safe cancer treatment and a continuum of care in most patients [6]. Later on, baseline characteristics and outcomes of cancer patients infected with SARS-CoV-2 were analyzed in the Lombardy region (Italy), highlighting the fact that patients should avoid exposure or increase their protection against SARS-CoV-2 while treatment adjustments and prioritizing vaccination should adequately be considered [7].

An important vulnerability that can the exploited by COVID-19 is the immunosuppressive condition provoked by both the cancer and antitumor therapies. Even though SARS-CoV-2 infection may be merely severe in any adults, older adults (65 years old or older) may experience a higher mortality rate because of their depressed immune status [8]. The same applies to individuals with coexisting comorbidities including hypertension, obesity, diabetes, or cancer [9] whose immune system cannot efficiently combat the SARS-CoV-2 infection [10]. Immune checkpoint blockade therapies with immune-checkpoint inhibitors (ICI) such as anti-PD-L1 (programmed cell death ligand 1) and anti-CTLA-4 (Cytotoxic T lymphocyte-associated antigen) have been investigated to potentiate T cell immunity in cancer patients. The binding of PD-L1 to its receptor PD-1 on activated T cells indeed attenuates anti-tumor immunity by inhibiting T cell-activating signals, and therefore, the functions of PD-1-positive T cells can be partially recovered with PD-1/PD-L1 blockade. The introduction of ICI in 2011 has revolutionized the management of many solid cancers and hematological malignancies [11]. In Gambichler’s perspectives, the authors present the pros and cons of using ICI in cancer patients with respect to the risk of acquiring an infection by SARS-CoV2 and mortality from COVID-19. They conclude that anti-PD-1/PD-L1- and/or anti-CTLA-4-based cancer treatment do not appear to increase vulnerability for SARS-CoV-2 infection per se. However, whether the use of ICI in cancer patients with COVID-19 increases morbidity and mortality related to SARS-CoV-2 infection cannot be definitively answered at this time [12]. Based on the positive effect that ICIs have on T-cell activity against cancer cells, as well as against virus-infected cells, ICIs administration may not only represent a risk for cancer patients during this pandemic but can be protective for cancer patients undergoing SARS-CoV-2 infection, according to Vivarelli et al. [10]. Moreover, in the Goshen-Lago et al. study, the authors suggest that, due to the differential immune cell profile of cancer patients who are treated with immunomodulatory agents, the host response to the SARS-CoV-2 may lessen symptom severity [13].

In the review by Allegra et al., the authors highlight some diagnostic and therapeutic challenges by analyzing some lesser-known aspects of the relationship between neoplasms and SARS-CoV-2 infection. They started analyzing the different expressions of the ACE2 receptor utilized by the virus in the various neoplastic pathologies, and the roles that different cytokine patterns could have in vulnerability to infection and the appearance of complications. They also suggest the possible use of drugs commonly employed in neoplastic therapy, such as bevacizumab, ibrutinib, elinexor, thalidomide, carfilzomib, and PD-1 inhibitors, for the treatment of SARS-CoV-2 infection [14]. Delivering cytotoxic chemotherapy during a pandemic has been challenging, and both oncologists and patients have been concerned that chemotherapy-induced immunosuppression could increase the risk of unfavorable outcomes from COVID-19. Some findings data do not suggest active chemotherapy as an independent risk factor or death from COVID-19, although the effects of different primary tumors and cytotoxic drugs on COVID-19 outcomes should be further investigated [15]. The French society of pediatric oncology (SFCE) analyzed the relationship between COVID-19 and pediatric oncology, showing that, unlike the elderly, relatively few pediatric cancer patients had clinical signs of COVID-19 or were infected by SARS-CoV-2, although, even in this study, some patients were highly immunocompromised and at risk of developing severe forms of COVID-19 [16].

The outcomes of COVID-19 in patients with comorbidities and amongst minorities have also been analyzed, showing, for instance, that elevated white blood cell counts, especially neutrophils and high C-reactive protein (CRP) levels at admission, were associated with an increased requirement of orotracheal intubation (OTI) [17] and that men older than 70 years of age had a greater than four-fold increase in odds of dying or being transitioned to hospice care compared to females and those ≤70 years of age [18]. Using data derived from a Korean nationwide longitudinal cohort of 5628 COVID-19 patients, the authors show that lymphopenia may serve as a reliable predictive factor for COVID-19 clinical outcomes [19]. Interestingly, the implications for cancer patients, who can receive hormonal therapies during their treatment plans, have been also analyzed in the presence of COVID-19. Sex hormones are key actors in the age-dependent and sex-specific severity of COVID-19, through several mechanisms, such as regulation of the immune responses, modulation of SARS-CoV-2 cell entry, and hemostatic function. The authors report a potential protective role of female hormones, whereas conflicting results are reported regarding androgens, although further investigations are needed [20]. Other studies focused on the care experiences and health-related quality of life (HRQoL) in sarcoma patients during the COVID-19 pandemic [21] and analyzed also the psychological impact of the COVID-19 pandemic on healthcare providers [22]. Finally, it is still unknown whether survivors of COVID-19-infected subjects are at higher risk for developing cancer and whether any biologic and clinical features exist in post-COVID-19 individuals that might be related to carcinogenesis. The induction of a cytokine storm may facilitate pulmonary fibrosis development that may reflect the appearance of Ground Glass Opacity (GGO) in the lungs of COVID-19 patients. GGO nodules, if persisting in COVID-19 patients, might potentially transform into lung cancer in the presence of other specific conditions such as smoking and certain genetic susceptibility [23].

In the second part of this series, studies mainly focused on analyses of large cohorts of patients with respect to diagnostic, therapeutic, and prognostic purposes. Particular focus was directed to the delayed management of cancer screening, which occurred almost worldwide. For instance, it has been shown in a study in Germany that the number of new cancer diagnoses significantly decreased between March and May 2020 compared with 2019, and this was not because of a sudden decline in the actual incidence of cancer but likely because many cases had been undiagnosed or diagnosed with some delay. This outcome highlighted the need for public health measures aimed at improving the management of cancer during the ongoing pandemic in Germany [24,25]. A reduction in cancer screening was also shown by analyzing data from the 2014–2020 Behavioral Risk Factor Surveillance System (BRFSS) survey in the United States, with a specific reduction in screening in 2020 compared to screening percentages in 2014–2019 [26]; by the experience of the Pathology Department of the Fondazione IRCCS Istituto Nazionale Tumori (INT) in Milan (Italy), highlighting how the sharp slowdown in cancer screening during the first wave of COVID-19 could seriously endanger cancer prevention in the near future [27]; by analyzing all the patients with a pathological diagnosis of cancer attended to in two hospitals in Málaga (Spain) during the first year of pandemic compared with the patients diagnosed during the previous year 2019 [28]; and by analyzing the outcomes of the COVID-19 pandemic resulting from delayed diagnosis, staging, and treatment of cancer [29,30,31].

Other studies focused on the safety of both oncologic patients and medical and paramedical staff through the development of management protocol [32] and monitoring positive patients [33]. In April 2020 at Fondazione IRCCS Istituto Nazionale dei Tumori, Milan (Italy), one of the three oncologic hubs in Lombardy, the authors implemented a prospective longitudinal study aimed at monitoring the serological response to SARS-CoV-2 in healthcare personnel (HCP) in order to prevent the spread of infection among patients and medical staff, waiting for the effectiveness of the active immunization by SARS-CoV-2 vaccines [34]. Regarding the lack of treatment for COVID-19 infections in cancer patients, Mustafa et al. report the effect of Rintatolimod, a Toll-like receptor 3 (TLR3) agonist, on human epithelial cancerous cells. They show that Rintatolimod stimulated an anti-viral effect by producing RNase L that blocks virus replication. Moreover, Rintatolimod activated the innate and the adaptive immune systems by activating a cascade of actions in human cancerous cells, suggesting that Rintatolimod should be considered in the treatment regimens of cancer patients infected by SARS-CoV-2 [35].

We hope readers enjoy this Special Issue of *Cancers* and that it will help readers to understand the impact that COVID-19 had, has, and will have on cancer. We also hope that these findings will ignite clinical research aimed at finding anti-cancer drugs able to minimize the risk of COVID-19 infection and improve cancer treatment and prognosis in the presence of COVID-19 infection.

## Data Availability

Not applicable.
